# Myo-Inositol and D-Chiro-Inositol Reduce DHT-Stimulated Changes in the Steroidogenic Activity of Adult Granulosa Cell Tumors

**DOI:** 10.3390/ijms252010974

**Published:** 2024-10-12

**Authors:** Anna Maria Wojciechowska, Paulina Zając, Justyna Gogola-Mruk, Magdalena Karolina Kowalik, Anna Ptak

**Affiliations:** 1 Department of Human Physiology and Pathophysiology, School of Medicine, Collegium Medicum, University of Warmia and Mazury, 10-082 Olsztyn, Poland; 2 Department of Physiology and Toxicology, Institute of Animal Reproduction and Food Research of the Polish Academy of Sciences, 10-747 Olsztyn, Poland; p.zajac@pan.olsztyn.pl (P.Z.); m.kowalik@pan.olsztyn.pl (M.K.K.); 3 Laboratory of Physiology and Toxicology of Reproduction, Institute of Zoology and Biomedical Research, Jagiellonian University, 30-387 Krakow, Poland; justyna.gogola@uj.edu.pl (J.G.-M.); anna.ptak@uj.edu.pl (A.P.)

**Keywords:** adult granulosa cell tumors, D-chiro-inositol, dihydrotestosterone, myo-inositol, steroidogenesis

## Abstract

Considering the properties of myo-inositol (MI) and D-chiro-inositol (DCI), which are well known in polycystic ovary syndrome therapy, and the limitations of adult granulosa cell tumor (AGCT) treatment, especially for androgen-secreting tumors, we studied the role of MI and DCI in the androgen-rich environment of AGCTs. For this purpose, we analyzed the mRNA expression of steroidogenic genes and the secretion of progesterone (P4) and 17β-estradiol (E2) in an unstimulated and/or dihydrotestosterone (DHT)-stimulated environment under MI and DCI influence. Thus, we used the HGrC1 and KGN cell lines as in vitro models of healthy and cancerous granulosa cells. We found that DHT, the most potent androgen, increased E2 secretion and steroidogenic acute regulatory protein (*StAR*) and cytochrome P450 side-chain cleavage gene (*CYP11A1*) mRNA expression without affecting 450 aromatase (*CYP19A1*) in AGCTs. However, after the MI and DCI treatment of KGN cells, both compounds strongly reduced *StAR* and *CYP11A1* expression. Interestingly, in DHT-stimulated KGN cells, only DCI alone and its cotreatment with MI reduced both *CYP11A1* mRNA and E2 secretion. These findings suggest that *CYP11A1* is responsible for the antiestrogenic effect of DCI in the androgen-rich environment of AGCTs. Therefore, MI and DCI could be used as effective agents in the adjuvant treatment of AGCT, but further detailed studies are needed.

## 1. Introduction

Granulosa cell tumors (GCTs) represent 5% of all ovarian cancers; however, they are the most common subtype of ovarian sex cord-stromal tumors. Among them, adult granulosa cell tumors (AGCTs) are more common and account for approximately 95% of all GCTs. AGCT is an endocrine ovarian cancer, and its unique feature is the ability to synthesize estrogens and express steroid hormones [1]. However, a small subset of cases are androgenic, accounting for <3% of AGCTs [2]. Moreover, androgen/androgen receptor (AR) signaling has been found to promote tumorigenesis and metastasis in several cancer types, including AGCT [3]. Its survival outcomes are generally favorable; however, in 20–30% of patients, the disease relapses within 5 years from diagnosis, with 50% leading to death [4].

AGCT is characterized by a mutation in the forkhead box protein L2 (*FOXL2*) gene, which leads to overactivation of steroidogenesis [5,6]. It was found that FOXL2C134W is able to enhance 450 aromatase (*CYP19A1*) mRNA expression together with estrogen concentration increase, which is one of the mechanisms explaining the excessive estrogen levels in women with AGCTs [7]. The therapeutic options for recurrent AGCTs are still limited; however, due to AGCTs having the hormone synthesis function of normal GCs, hormonal signaling could serve as a potential therapeutic target for the treatment of this disease.

Myo-inositol (MI) and D-chiro-inositol (DCI) are plant-derived polyols found in almost every human tissue and are involved in many cellular processes [8,9,10]. The use of MI and DCI in the treatment of polycystic ovary syndrome (PCOS) is well known [11,12,13]. Moreover, because of their influence on steroidogenesis, both isomers have been examined for the treatment and prevention of breast cancer [14,15] and for other estrogen-sensitive pathologies, such as endometrial hyperplasia and endometriosis [16]. Importantly, MI can decrease androgen synthesis and increase the expression of *CYP19A1*. In turn, DCI downregulates the expression of *CYP19A1* [16] and the cytochrome P450 side-chain cleavage gene (*CYP11A1*) while increasing androgen production [17]. These compounds may therefore be effective in hormone-dependent cancers by regulating hormone homeostasis. However, there is a lack of research on the influence of MI and DCI on the processes that occur in AGCTs.

In fact, an androgenic environment can induce the growth and development of AGCTs or increase side effects, especially in patients with hyperandrogenism, and MI and DCI can regulate hormonal steroidogenesis [3,18]. In the present study, we investigated the role of MI and DCI in the presence of an androgen-rich environment in AGCTs. For this purpose, we tested the mRNA expression of steroidogenic acute regulatory protein (*StAR*), *CYP11A1*, and *CYP19A1* as well as progesterone (P4) and 17β-estradiol (E2) secretion in unstimulated and dihydrotestosterone (DHT)-stimulated environments. We chose DHT because it binds to the AR with higher affinity than testosterone (T). Moreover, its biological activity exceeds that of T by up to 10 times, which makes DHT the primary ligand for AR [19]. These studies were conducted using two GC cell lines, HGrC1 and KGN, which are good in vitro models for understanding the cellular mechanisms of human healthy ovarian granulosa cells and recurrent granulosa cell tumors.

## 2. Results

### 2.1. AGCTs Are More Sensitive to Androgen Action than Noncancer Granulosa Cells

**Figure 1:**
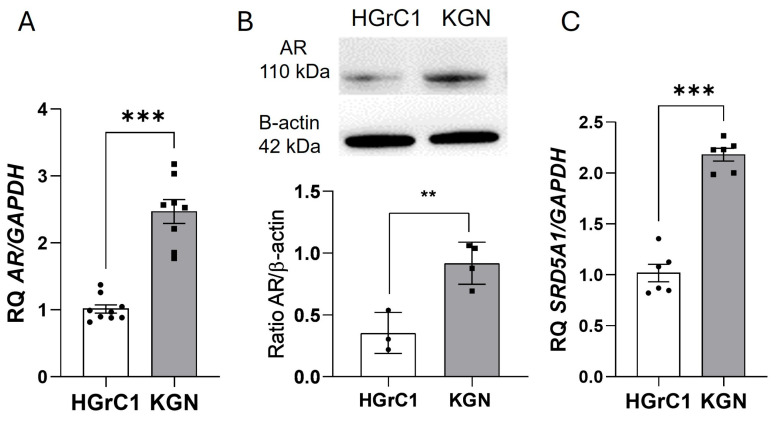
Basal mRNA (**A**) and protein (**B**) expression of AR and mRNA expression of *SRD5A1* (**C**) in HGrC1 and KGN cells. The relative expression (RQ) of HGrC1 was set to 1. The data represent the mean ± SEM of three independent experiments. ** *p* < 0.01, *** *p* < 0.0001.

To determine the effect of androgens on AGCTs, we performed a comparison between the HGrC1 and KGN cell lines. We found that the mRNA and protein expression levels of AR were higher (2.5-fold and 2.62-fold, respectively) in KGN than in HGrC1 cells (Figure 1A,B). Moreover, mRNA expression of 5α-reductase (*SRD5A1*), which converts T into the more active metabolite DHT, was higher (2.14-fold) in the cell-like tumor cell line (Figure 1C).

### 2.2. KGN Cell Viability Is Not Affected by DHT

**Figure 2:**
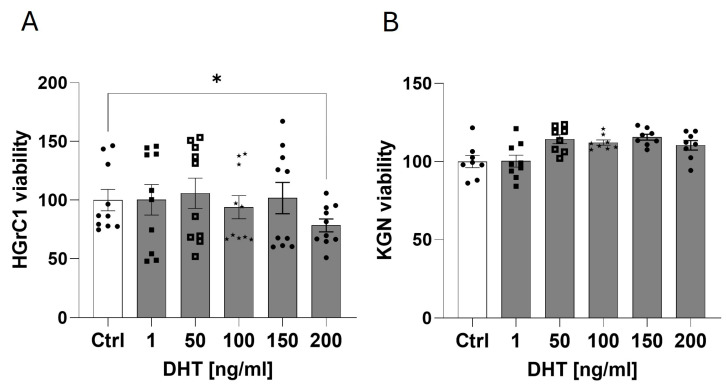
Effect of DHT at concentrations of 1, 50, 100, 150, and 200 ng/mL on the viability of HGrC1 (**A**) and KGN (**B**) cells after 48 h of incubation. The data represent the mean ± SEM of three independent experiments. Ctrl, control. * *p* < 0.05.

Because KGN cells exhibited markedly higher *AR* expression than HGrC1, we determined the viability of both HGrC1 and KGN cells in response to DHT at concentrations of 1, 50, 100, 150, and 200 ng/mL. Among the HGrC1 cells, DHT at a concentration of 200 ng/mL significantly reduced cell viability (Figure 2A). However, DHT did not increase KGN cell viability (Figure 2B) at any of the tested doses. DHT at a dose of 150 ng/mL (500 nM) seems to be the highest concentration at which it did not reduce HGrC1 cell viability; thus, we used this dose for further experiments.

### 2.3. Steroidogenic Enzymes Are Highly Expressed in AGCTs

**Figure 3:**
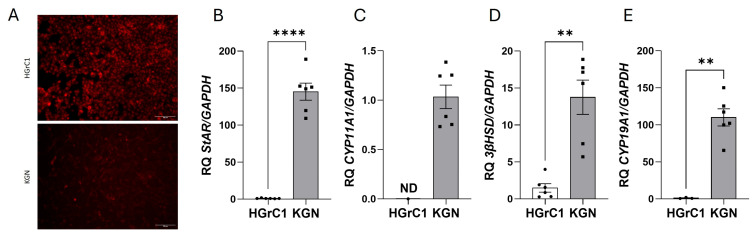
Lipid content in HGrC1 and KGN cells stained with Nile Red dye, scale bar = 100 µm (**A**) and basal mRNA expression of *StAR* (**B**), *CYP11A1* (**C**), *3β-HSD* (**D**), and *CYP19A1* (**E**) in both cell lines. The relative expression (RQ) of HGrC1 was set to 1. The data represent the mean ± SEM of three independent experiments. ** *p* < 0.01, **** *p* < 0.0001.

To visualize lipid droplets, HGrC1 and KGN cells were stained with Nile Red dye. We detected more intracellular lipids in HGrC1 than in KGN cells (Figure 3A). We also demonstrated that HGrC1 expressed *StAR*, 3β-hydroxysteroid dehydrogenase (*3β-HSD*), and *CYP19A1* (Figure 3B,D,E); however, these genes were expressed at markedly greater levels (145.2-fold for *StAR*, 13.8-fold for *3β-HSD*, 110.2-fold for *CYP19A1*) in KGN cells (Figure 3B,D,E). In HGrC1 cells, *CYP11A1* mRNA was not expressed, whereas it was expressed in KGN cells (Figure 3C).

### 2.4. DHT Induces E2 Secretion in KGN Cells by Affecting the Expression of Steroidogenic Enzymes

**Figure 4:**
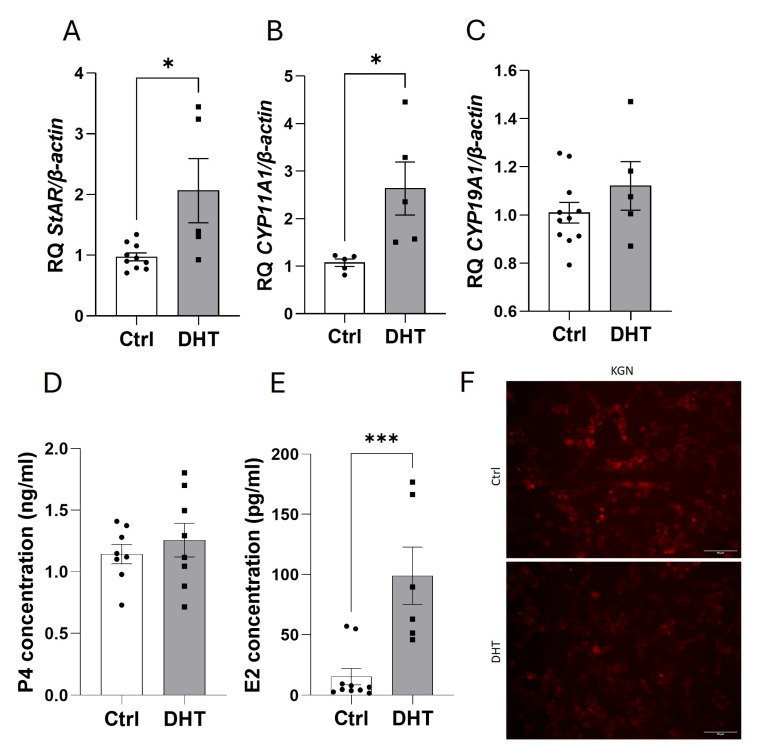
Effects of DHT (500 nM) on *StAR* (**A**), *CYP11A1* (**B**), and *CYP19A1* (**C**) mRNA expression as well as P4 (**D**) and E2 (**E**) secretion and lipid content determined via Nile Red staining, scale bar = 50 µm (**F**) after incubation of KGN cells for 24 h. The relative expression (RQ) of the Ctrl was set to 1. The data represent the mean ± SEM of three or four independent experiments. Ctrl, control. * *p* < 0.05, *** *p* < 0.001.

Next, we tested the mRNA expression of steroidogenic enzymes under the influence of DHT. We found that DHT (500 nM) increased the mRNA expression of *StAR* (2.1-fold) (Figure 4A) and *CYP11A1* (2.6-fold) (Figure 4B) without affecting P4 secretion (Figure 4D). The increased mRNA expression of both enzymes was confirmed by decreased levels of intracellular lipids in DHT-stimulated KGN cells (Figure 4F). Moreover, we observed an increase in the E2 concentration (6.5-fold) (Figure 4E) in response to DHT; however, *CYP19A1* mRNA expression remained unchanged (Figure 4C).

### 2.5. Neither Mio-Inositol nor D-Chiro-Inositol Affects the Viability of HGrC1 or KGN Cells

**Figure 5:**
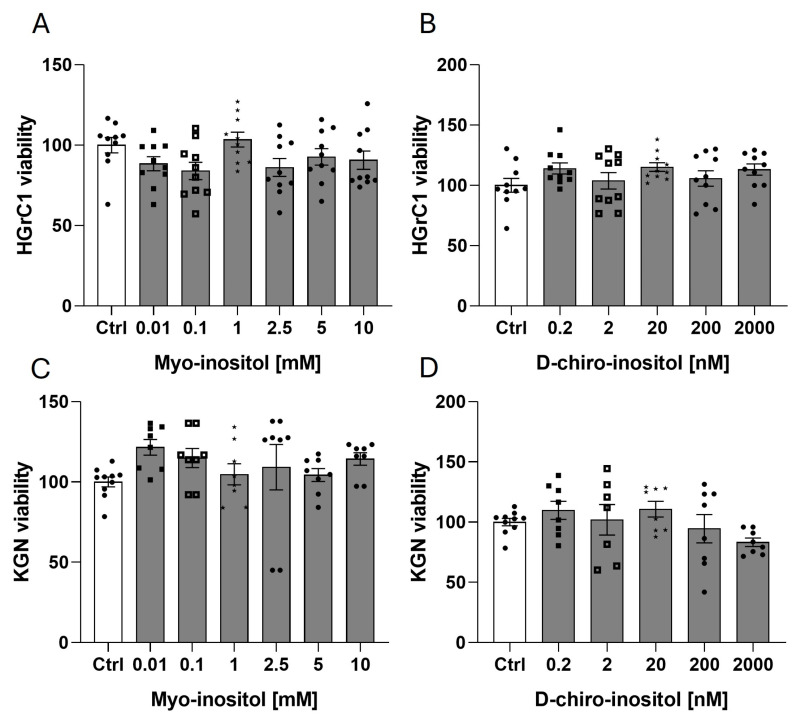
Effects of MI (0.01, 0.1, 1, 2.5, 5, and 10 mM) and DCI (0.2, 2, 20, 200, and 2000 nM) on the viability of HGrC1 (**A**,**B**) and KGN (**C**,**D**) cells after 48 h of incubation. The data represent the mean ± SEM of three independent experiments. Ctrl, control.

To determine the optimal treatment dose before MI and DCI are used, we examined HGrC1 or KGN cell viability after incubation with MI at concentrations of 0.01, 0.1, 1, 2.5, 5, and 10 mM and DCI at concentrations of 0.2, 2, 20, 200, and 2000 nM for 48 h. MI and DCI did not affect HGrC1 (Figure 5A,B) or KGN cell viability (Figure 5C,D); thus, middle doses of 1 mM for MI and 20 nM for DCI were chosen for further experiments.

### 2.6. MI and DCI Strongly Reduce Steroidogenesis in Adult GC Tumors

**Figure 6:**
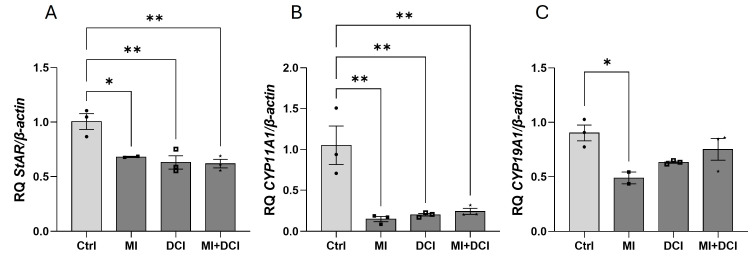
Effects of MI (1 mM) and DCI (20 nM), added alone or together, on *StAR* (**A**), *CYP11A1* (**B**), and *CYP19A1* (**C**) mRNA expression after incubation of KGN for 24 h. The relative expression (RQ) of the Ctrl was set to 1. The data represent the mean ± SEM of three independent experiments. Ctrl, control. * *p* < 0.05, ** *p* < 0.01.

Inositols are involved in the regulation of ovarian steroidogenesis; however, their role in relation to AGCTs has not been studied. Thus, KGN cells were incubated for 24 h with MI (1 mM), DCI (20 nM), and both MI (1 mM) and DCI (20 nM) administered together. We found that MI and DCI alone and in combination reduced the mRNA expression of *StAR* (1.4-fold for MI, 1.7-fold for DCI, 1.7-fold for both) (Figure 6A) and *CYP11A1* (6.7-fold for MI, 5-fold for DCI, 4.2-fold for both) (Figure 6B), while *CYP19A1* mRNA expression was significantly reduced by MI (2-fold) (Figure 6C).

### 2.7. Cotreatment with MI and DCI Reduces DHT-Stimulated Steroid Secretion via Steroidogenic Enzymes

**Figure 7:**
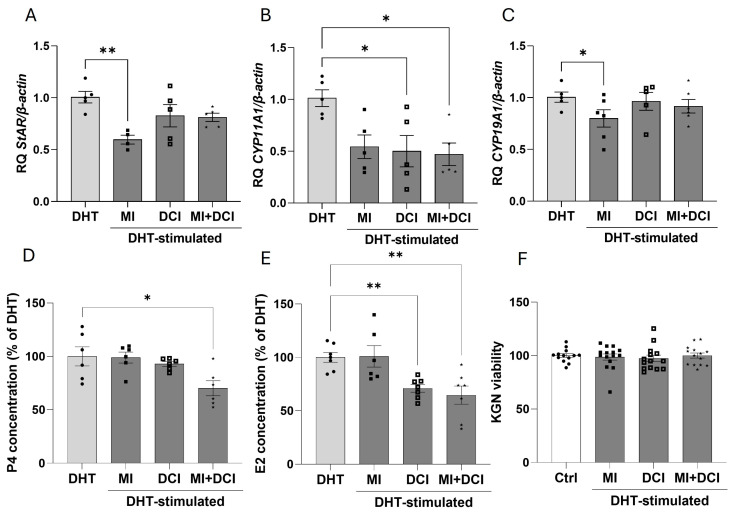
Effects of MI (1 mM) and DCI (20 nM), added alone or together, on *StAR* (**A**), *CYP11A1* (**B**), and *CYP19A1* (**C**) mRNA expression; P4 (**D**) and E2 secretion (**E**); and cell viability (**F**) in DHT-stimulated KGN cells after incubation for 24 h. The relative expression (RQ) of DHT was set to 1. The data represent the mean ± SEM of three or four independent experiments. DHT, dihydrotestosterone used as a control. * *p* < 0.05, ** *p* < 0.01.

Next, we studied the effects of MI (1 mM) and DCI (20 nM) after the pre-stimulation of KGN with DHT (500 nM). We demonstrated that only MI significantly decreased the mRNA expression of *StAR* (1.7-fold) (Figure 7A), while DCI and cotreatment MI with DCI reduced the mRNA expression of *CYP11A1* (2-fold for DCI, 2.15-fold for both) (Figure 7B) in DHT-stimulated KGN cells. P4 secretion was decreased by cotreatment MI with DCI (1.4-fold) (Figure 7D), which appeared to be the result of changes in the expression of *StAR* and *CYP11A1*. We observed a decrease in *CYP19A1* mRNA expression (1.25-fold) (Figure 7C) only after MI stimulation, whereas E2 was reduced as a result of DCI addition and cotreatment MI with DCI (2-fold for DCI, 2.1-fold for both) (Figure 7E). These data indicate that *CYP19A1* is not involved in the changes in E2 secretion induced by MI or DCI in AGCTs. All the treatments used in this study did not affect KGN cell viability (Figure 7F).

## 3. Discussion

Although GCTs are thought to have a better prognosis than epithelial tumors, their adult subtype is characterized by frequent recurrence or metastasis after the removal of the primary tumor. Most GCTs (approximately 70%) are hormonally active and secrete estrogens, and 15% are hormonally neutral, while 10% can produce androgens [20]. Steroidogenesis is dysregulated in AGCTs, leading to increased estrogen synthesis via the direct induction of aromatase. Although in the adult subtype, less than 3% of tumors are androgen active, the importance of considering testosterone-secreting ovarian tumors has been highlighted in new case reports [21,22,23]. Our research shows for the first time that DHT leads to an increase in E2 synthesis in the AGCT cell line and that MI and DCI, known for their therapeutic effects, can be used in AGCT patients to reduce abnormal steroid secretion.

We compared two GC cell lines, a noncancerous HGrC1 cell line derived from GCs of antral follicles and a KGN cell line derived from recurrent adult ovarian granulosa cell tumors. We demonstrated that the expression of steroidogenic enzymes was markedly higher in KGN cells than in HGrC1 cells. This finding is consistent with the definition of this type of cancer, where a mutation in *FOXL2* specific to AGCTs causes changes in steroidogenesis, maintaining the female phenotype in granulosa cells [24]. Interestingly, we observed the overexpression of *AR* in KGN cells compared to HGrC1 cells, which is characteristic of AGCT tumors [25] and other AR-positive cancers. It is known that androgens can affect breast carcinogenesis by aromatization to estrogens or directly through AR [26,27]. We found that *SRD5A1* is also highly expressed in AGCTs. The *SRD5A1* gene encodes one of three 5α-reductase enzymes that metabolize T to the more active DHT in target tissues. Elevated levels of 5α-reductase mRNA were also observed in the granulosa cells of PCOS women; thus, the harmful effects of hyperandrogenism in the ovary may result from the conversion to reduced metabolites, including DHT [28]. As a result, AGCTs can easily be misdiagnosed as PCOS [21]. Therefore, in patients with abnormally elevated testosterone levels, clinicians should still be alert to the presence of these ovarian tumors. In addition, high *SRD5A1* expression in AGCTs, together with high *AR* expression, indicates high sensitivity to androgens, especially the most potent androgen, DHT.

Interestingly, DHT increased E2 secretion and the mRNA expression of *StAR* and *CYP11A1* in the AGCT cell line. In our studies, DHT could not act as a substrate because it is not converted into E2 like other androgens. However, published data indicate the involvement of androgens, including DHT, in stimulating lipid uptake, synthesis, storage, and lipolysis from lipid droplets and, in this way, regulating gene expression [25,29,30]. Moreover, DHT is directly involved in the regulation of lipid metabolism and the promotion of LDL effects in prostate cancer cells [31]. Therefore, DHT may lead to an increase in the E2 concentration in AGCTs through its influence on the amount and metabolism of cholesterol without affecting *CYP19A1*. Moreover, in our studies, the lower intracellular lipid concentration in AGCTs indicated that the substrate at the initial stage of steroidogenesis was continuously metabolized, which was consistent with the high expression of *StAR* and *CYP11A1*. On the other hand, the upregulation of *StAR* and *CYP11A1* in AGCTs could be a feedback effect of DHT-increased E2 concentration. In studies by Doblado et al., DHT was shown to stimulate the E2 concentration, and in turn, E2 was able to stimulate *CYP11A1* in rat GCs [32]. As our studies showed, in a DHT-stimulated environment, E2 concentrations in AGCTs are high, which increases the risk of undesirable effects caused not only by androgens but also by E2.

In these studies, we demonstrated that MI and DCI inhibited steroidogenesis in AGCTs by reducing *StAR* and especially *CYP11A1* expression. Both inositol stereoisomers are well known for their ability to treat PCOS; however, due to their broad effects, they are being investigated for use as supportive treatments for other metabolic or hormone-dependent diseases [33,34,35]. Interestingly, we did not observe the opposite effect of MI or DCI on steroidogenic gene expression in AGCTs that was observed in GCs [36]. However, under androgenic conditions, which we have achieved by stimulating cells with DHT, the action of both isomers changes. Thus, after stimulation with DHT, the mRNA expression of *StAR* is reduced only by MI alone, while that of *CYP11A1* is reduced by DCI alone and together with MI. As a result, we observed the inhibitory effect of DCI and DCI with MI on E2 secretion in DHT-stimulated KGN cells. We expected that DCI, which has antiestrogenic properties [37], would cause the inhibition of *CYP19A1*. Interestingly, our results suggested that *CYP11A1* expression is essential for the antiestrogenic effect of DCI in the androgen-rich environment of AGCTs. Several studies have shown that during ovarian steroidogenesis, DCI regulates steroidogenic enzymes, reducing the mRNA expression of both *CYP19A1* and *CYP11A1* in a dose–response manner [17]. Notably, simultaneous administration of MI and DCI could enhance follicle-stimulating hormone (FSH) and estrogen responsiveness [36].

In summary, our studies proved that the production of steroidogenic enzymes is at a higher level in AGCTs than in primary granulosa cells. Moreover, DHT increases the initial level of steroidogenic enzymes and thus the E2 concentration in AGCTs, which may cause side effects from both E2 and androgens. Approximately 26–38% of patients present with endometrial hyperplasia, and approximately 10% of patients are diagnosed with concurrent endometrial cancer due to long-term exposure to endogenous, abnormal estrogen [6]. Moreover, the MI and DCI results on steroidogenic activity in AGCTs indicate that the application of both agents during treatment is a good option for restoring physiological hormone levels. However, the use of DCI alone or in combination with MI would be particularly beneficial in AGCTs with high levels of androgen. A limitation is that this study included only cell lines; therefore, further large studies are warranted to build on these baseline data. Hence, the use of inositols as an adjuvant strategy requires further research to avoid possible undesired effects.

## 4. Materials and Methods

### 4.1. Treatments

An initial dose of human DHT was dissolved in methanol, whereas subsequent doses of DHT as well as all doses of MI and DCI were dissolved in redistilled water. All materials used in these studies were purchased from Merck Life Science (Rockville, MD, USA) unless otherwise stated.

### 4.2. Cell Line Culture

Experiments were performed using the human nonluteinized ovarian granulosa cell line HGrC1, which was a gift from Dr. Ikara Iwase (Nagoya University, Nagoya, Japan), and an ovarian adult granulosa cell tumor KGN cell line (RBRC-RCB1154; Riken Cell Bank, Ibaraki, Japan). The presence of the FOXL2 C134W mutation in the KGN cell line is consistent with mutant protein in the AGCT [38]. The HGrC1 cell line was cultured in phenol red-free DMEM (Sigma Aldrich, St. Louis, MO, USA) supplemented with 2 mM L-glutamine, whereas KGN cells were cultured in phenol red-free DMEM-F12 (Thermo Fisher Scientific, Waltham, MA, USA). Both media were supplemented with 10% charcoal-stripped fetal bovine serum (FBS; Biowest, Bradenton, FL, USA) under a controlled atmosphere (95% O_2_ + 5% CO_2_) and a temperature of 37 °C.

### 4.3. Cell Treatments

To investigate the viability of the HGrC1 and KGN cells, they were incubated with DHT at concentrations of 1, 50, 100, 150, or 200 ng/mL; MI at concentrations of 0.01, 0.1, 1, 2.5, 5, or 10 mM; and DCI at concentrations of 0.2, 2, 20, 200, or 2000 nM for 48 h. As a result, DHT at a dose of 150 ng/mL (500 nM), MI at a dose of 1 mM, and DCI at a dose of 20 nM were selected for further experiments.

KGN cells (*n* = 4) were plated in 6-well plates at 750,000/well and incubated in DMEM/Ham’s F-12 medium with 10% FBS in a controlled atmosphere. After changing the medium to DMEM/Ham’s F-12 with the addition of 0.1% (Bovine Serum Albumin) BSA, KGN cells were pre-stimulated with DHT for 24 h. Then, the cells were stimulated with MI and DCI alone and together. After 24 h of incubation, the media were collected and stored at −20 °C while the cells were covered with 500 µL of fenozol and stored at −80 °C until hormone evaluation and mRNA expression analysis, respectively.

### 4.4. Cell Viability

HGrC1 and KGN cell viability was determined using the Cell Proliferation Kit MTT test (Roche, Basel, Switzerland) and PrestoBlue^®^ Cell Viability Reagent (Thermo Fisher Scientific, Waltham, MA, USA) according to the manufacturer’s protocols.

### 4.5. Nile Red Staining

After 48 h of incubation with DHT (500 nM), HGrC1, and KGN cells were stained with Nile Red dye (Invitrogen, Carlsbad, CA, USA) for 30 min to reveal intracellular lipids. A stock solution of dye was diluted 1:100 in medium without FBS. Then, the cells were examined using an Axiocam 503 (Zeiss, Jenoptik, Germany) bright-field fluorescence microscope (excitation wavelength: 590 nm).

### 4.6. Hormone Determination

The secretion of E2 and P4 levels in the media were determined using an enzyme-linked immunosorbent assay (ELISA). Horseradish peroxidase-labelled E2 and P4 were used as tracers. The standard curve range and the intra- and inter-assay coefficients of variation were as follows: E2—6.25–1600 pg/mL, 9.9%, and 9.9%; P4—0.1–25 ng/mL, 14.8%, and 9.8%. The method’s sensitivity was 0.15 ng/mL for P4 and 1.5 pg/mL for E2. All assays were performed in 96-well plates that were coated with ovine anti-rabbit γ-globulin, which was obtained in the Department of Physiology and Toxicology of Reproduction, Institute of Animal Reproduction and Food Research, Polish Academy of Science in Olsztyn. The absorbance was measured at a wavelength of 450 nm using an Epoch Microplate Spectrophotometer (BioTek Instruments, Charlotte, VT, USA). If the secretion values were below the detection level, the graphs were not included.

### 4.7. Real-Time PCR Analysis

The analysis of genes involved in the biosynthesis of steroid hormones was conducted using SYBR Green and TaqMan real-time PCR. After mRNA isolation using a Total RNA kit (A&A Biotechnology, Gdansk, Poland) and cDNA synthesis using a Maxima First Strand cDNA synthesis kit, SYBR Green real-time PCR analysis was performed with SYBR™ Green PCR Master Mix. TaqMan real-time PCR probes were obtained using a TaqMan Gene Expression Cells-to-CT Kit, a StepOne-Plus Real-time PCR system, and TaqMan gene expression assays in combination with TaqMan gene expression master mix containing the ROX passive reference dye. The expression of the studied genes was normalized using *β-actin* or *GAPDH* as reference genes. All primers used are listed in Table 1. All reagents for real-time PCR were purchased from Thermo Fisher Scientific (Waltham, MA, USA). Duplicate control samples lacking cDNA were prepared for each gene, and no amplification was detected. All the results were calculated using the 2^−ΔΔCt^ method [39].

### 4.8. Western Blot Analysis

Antibodies against the androgen receptor (ab9474; Abcam, Cambridge, UK) and an anti-mouse secondary antibody (#7076; Cell Signaling Technology, Danvers, MA, USA) were used to analyze the expression of the AR protein in both the HGrC1 and KGN cell lines. An antibody against β-actin (A5316) was used as a loading control. Western blot analysis was performed as described previously [40].

### 4.9. Statistical Analysis

The data are presented as the mean ± SEM of at least three independent experiments. Statistical analysis was performed using one-way ANOVA followed by Tukey’s multiple comparison test or the nonparametric Student’s *t* test (GraphPad 8 Software, La Jolla, CA, USA). The level of significance was set at * *p* < 0.05, ** *p* < 0.01, *** *p* < 0.001, and **** *p* < 0.0001.

## Figures and Tables

**Table 1:** Primers used for SYBR Green and TaqMan real-time PCR.

Gene	SYBR Green Primers	TaqMan Primers
*StAR*	R-5′tgcgctggcagtacatgtg3′; F-5′gctgcttgttctgtggtgttg3′	Hs00986559_g1
*CYP11A1*	R-5′ccctctcctggtgacaatgg3′; F-5′tgacataaaccgactccacgtt3′	Hs00167984_m1
*CYP19A1*	R-5′gatagcagaaaaaagacgcaggat3′;F-5′ttagggtgctttgcaatgagaa3′	Hs00903411_m1
*β-actin*	R-5′tcctccctggagaagagct3′; F-5′tttcgtggatgccacaggat3′	-
*3βHSD*	-	Hs04194787_g1
*AR*	-	Hs00171172_m1
*SRD5A1*	-	Hs00971645_g1
*GAPDH*	-	4310884E

## Data Availability

The datasets presented in this article are not readily available because the data are part of an ongoing study. Requests to access the datasets should be directed to the corresponding author.

## References

[1] Haltia U.M., Pihlajoki M., Andersson N., Mäkinen L., Tapper J., Cervera A., Horlings H.M., Turpeinen U., Anttonen M., Bützow R. (2020). Functional Profiling of FSH and Estradiol in Ovarian Granulosa Cell Tumors. J. Endocr. Soc..

[2] Macut D., Ilić D., Mitrović Jovanović A., Bjekić-Macut J. (2019). Androgen-Secreting Ovarian Tumors. Front. Horm. Res..

[3] Summey R.M., Rader J.S., Moh M., Bradley W., Uyar D., Bishop E., McAlarnen L., Hopp E. (2022). A Case Series of Triplet Anti-Hormonal Therapy in Androgen Receptor-Positive Recurrent Adult Ovarian Granulosa Cell Tumor. Gynecol. Oncol. Rep..

[4] Färkkilä A., Haltia U.M., Tapper J., McConechy M.K., Huntsman D.G., Heikinheimo M. (2017). Pathogenesis and Treatment of Adult-Type Granulosa Cell Tumor of the Ovary. Ann. Med..

[5] Llano E., Todeschini A.L., Felipe-Medina N., Corte-Torres M.D., Condezo Y.B., Sanchez-Martin M., López-Tamargo S., Astudillo A., Puente X.S., Pendas A.M. (2023). The Oncogenic FOXL2 C134W Mutation Is a Key Driver of Granulosa Cell Tumors. Cancer Res..

[6] Li X., Tian B., Liu M., Miao C., Wang D. (2022). Adult-Type Granulosa Cell Tumor of the Ovary. Am. J. Cancer Res..

[7] Belli M., Iwata N., Nakamura T., Iwase A., Stupack D., Shimasaki S. (2018). FOXL2C134W-Induced CYP19 Expression via Cooperation with SMAD3 in HGrC1 Cells. Endocrinology.

[8] Yang L., Yang M., Cui C., Long X., Li Y., Dai W., Lang T., Zhou Q. (2023). The Myo-Inositol Biosynthesis Rate-Limiting Enzyme ISYNA1 Suppresses the Stemness of Ovarian Cancer via Notch1 Pathway. Cell Signal.

[9] Watkins O.C., Pillai R.A., Selvam P., Yong H.E.J., Cracknell-Hazra V.K.B., Sharma N., Cazenave-Gassiot A., Bendt A.K., Godfrey K.M., Lewis R.M. (2023). Myo-Inositol Alters the Effects of Glucose, Leptin and Insulin on Placental Palmitic Acid and Oleic Acid Metabolism. J. Physiol..

[10] Al-Suod H., Ligor M., Rațiu I.A., Rafińska K., Górecki R., Buszewski B. (2017). A Window on Cyclitols: Characterization and Analytics of Inositols. Phytochem. Lett..

[11] Genazzani A.D., Santagni S., Rattighieri E., Chierchia E., Despini G., Marini G., Prati A., Simoncini T. (2014). Modulatory Role of D-Chiro-Inositol (DCI) on LH and Insulin Secretion in Obese PCOS Patients. Gynecol. Endocrinol..

[12] Unfer V., Facchinetti F., Orrù B., Giordani B., Nestler J. (2017). Myo-Inositol Effects in Women with PCOS: A Meta-Analysis of Randomized Controlled Trials. Endocr. Connect.

[13] Greff D., Juhász A.E., Váncsa S., Váradi A., Sipos Z., Szinte J., Park S., Hegyi P., Nyirády P., Ács N. (2023). Inositol Is an Effective and Safe Treatment in Polycystic Ovary Syndrome: A Systematic Review and Meta-Analysis of Randomized Controlled Trials. Reprod. Biol. Endocrinol..

[14] Amabile M.I., De Luca A., Tripodi D., D’alberti E., Melcarne R., Imbimbo G., Picconi O., D’andrea V., Vergine M., Sorrenti S. (2021). Effects of Inositol Hexaphosphate and Myo-Inositol Administration in Breast Cancer Patients during Adjuvant Chemotherapy. J. Pers. Med..

[15] Monti N., Dinicola S., Querqui A., Fabrizi G., Fedeli V., Gesualdi L., Catizone A., Unfer V., Bizzarri M. (2023). Myo-Inositol Reverses TGF-Β1-Induced EMT in MCF-10A Non-Tumorigenic Breast Cells. Cancers.

[16] Gambioli R., Forte G., Aragona C., Bevilacqua A., Bizzarri M., Unfer V. (2021). The Use of D-Chiro-Inositol in Clinical Practice. Eur. Rev. Med. Pharmacol. Sci..

[17] Sacchi S., Marinaro F., Tondelli D., Lui J., Xella S., Marsella T., Tagliasacchi D., Argento C., Tirelli A., Giulini S. (2016). Modulation of Gonadotrophin Induced Steroidogenic Enzymes in Granulosa Cells by D-Chiroinositol. Reprod. Biol. Endocrinol..

[18] Bizzarri M., Monti N., Piombarolo A., Angeloni A., Verna R. (2023). Myo-Inositol and D-Chiro-Inositol as Modulators of Ovary Steroidogenesis: A Narrative Review. Nutrients.

[19] Otala M., Mäkinen S., Tuuri T., Sjöberg J., Pentikäinen V., Matikainen T., Dunkel L. (2004). Effects of Testosterone, Dihydrotestosterone, and 17-Estradiol on Human Ovarian Tissue Survival in Culture. Fertil. Steril..

[20] Adefris M., Fekadu E. (2017). Postmenopausal Mild Hirsutism and Hyperandrogenemia Due to Granulosa Cell Tumor of the Ovary: A Case Report. J. Med. Case Rep..

[21] Jiang Z., Qiu Y., Hu S., Li Y., Chen X., Jin Y., Dai H. (2023). Testosterone Elevation in Ovarian Adult Granulosa Cell Tumor: A Case Report and Review of the Literature. Medicine.

[22] Šepić T.S., Severinski N.S., Eminović S., Badovinac A.R., Višnić A. (2023). Complete Restoration of Fertility in a Patient Treated for Androgensecreting Granulosa Cell Tumor-Case Report. JBRA Assist. Reprod..

[23] Rajamani K., Moore R.G., Stanard S.M., Astapova O. (2022). Testosterone-Secreting Endometrioid Ovarian Carcinoma Presenting With Hyperandrogenism. AACE Clin. Case Rep..

[24] Uhlenhaut N.H., Jakob S., Anlag K., Eisenberger T., Sekido R., Kress J., Treier A.C., Klugmann C., Klasen C., Holter N.I. (2009). Somatic Sex Reprogramming of Adult Ovaries to Testes by FOXL2 Ablation. Cell.

[25] Butler L.M., Centenera M.M., Swinnen J.V. (2016). Androgen Control of Lipid Metabolism in Prostate Cancer: Novel Insights and Future Applications. Endocr.-Relat. Cancer.

[26] Majumder A., Singh M., Tyagi S.C. (2017). Post-Menopausal Breast Cancer: From Estrogen to Androgen Receptor. Oncotarget.

[27] Wu Y., Vadgama J.V. (2017). Androgen Receptor as a Potential Target for Treatment of Breast Cancer. Int. J. Cancer Res. Mol. Mech..

[28] Jakimiuk A.J., Weitsman S.R., Magoffin D.A. (1999). 5-Reductase Activity in Women with Polycystic Ovary Syndrome. J. Clin. Endocrinol. Metab..

[29] Swinnent J.V., Van Veldhoven P.P., Esquenet M., Heyns W., Verhoeven G. (1996). Androgens Markedly Stimulate the Accumulation of Neutral Lipids in the Human Prostatic Adenocarcinoma Cell Line LNCaP. Endocrinology.

[30] Tousignant K.D., Rockstroh A., Fard A.T., Lehman M.L., Wang C., McPherson S.J., Philp L.K., Bartonicek N., Dinger M.E., Nelson C.C. (2019). Lipid Uptake Is an Androgen-Enhanced Lipid Supply Pathway Associated with Prostate Cancer Disease Progression and Bone Metastasis. Mol. Cancer Res..

[31] Cardoso H.J., Figueira M.I., Carvalho T.M.A., Serra C.D.M., Vaz C.V., Madureira P.A., Socorro S. (2022). Androgens and Low Density Lipoprotein-Cholesterol Interplay in Modulating Prostate Cancer Cell Fate and Metabolism. Pathol. Res. Pract..

[32] Doblado M., Zhang L., Toloubeydokhti T., Garzo G.T., Chang R.J., Duleba A.J. (2020). Androgens Modulate Rat Granulosa Cell Steroidogenesis. Reprod. Sci..

[33] Dinicola S., Unfer V., Soulage C.O., Yap-Garcia M.I.M., Bevilacqua A., Benvenga S., Barbaro D., Wdowiak A., Nordio M., Dewailly D. (2024). D-Chiro-Inositol in Clinical Practice: A Perspective from the Experts Group on Inositol in Basic and Clinical Research (EGOI). Gynecol. Obstet. Investig..

[34] Fatima K., Jamil Z., Faheem S., Adnan A., Javaid S.S., Naeem H., Mohiuddin N., Sajid A., Ochani S. (2023). Effects of Myo-Inositol vs. Metformin on Hormonal and Metabolic Parameters in Women with PCOS: A Meta-Analysis. Ir. J. Med. Sci..

[35] Motuhifonua S.K., Lin L., Alsweiler J., Crawford T.J., Crowther C.A. (2023). Antenatal Dietary Supplementation with Myo-Inositol for Preventing Gestational Diabetes. Cochrane Database Syst. Rev..

[36] Fedeli V., Catizone A., Querqui A., Unfer V., Bizzarri M. (2023). The Role of Inositols in the Hyperandrogenic Phenotypes of PCOS: A Re-Reading of Larner’s Results. Int. J. Mol. Sci..

[37] Monastra G., Vazquez-Levin M., Bezerra Espinola M.S., Bilotta G., Laganà A.S., Unfer V. (2021). D-Chiro-Inositol, an Aromatase down-Modulator, Increases Androgens and Reduces Estrogens in Male Volunteers: A Pilot Study. Basic Clin. Androl..

[38] Jamieson S., Butzow R., Andersson N., Alexiadis M., Unkila-Kallio L., Heikinheimo M., Fuller P.J., Anttonen M. (2010). The FOXL2 C134W Mutation Is Characteristic of Adult Granulosa Cell Tumors of the Ovary. Mod. Pathol..

[39] Livak K.J., Schmittgen T.D. (2001). Analysis of Relative Gene Expression Data Using Real-Time Quantitative PCR and the 2^−ΔΔCT^ Method. Methods.

[40] Gogola J., Hoffmann M., Ptak A. (2019). Persistent Endocrine-Disrupting Chemicals Found in Human Follicular Fluid Stimulate the Proliferation of Granulosa Tumor Spheroids via GPR30 and IGF1R but Not via the Classic Estrogen Receptors. Chemosphere.

